# Bone morphogenetic protein 10 and one-year outcomes after open revascularisation in symptomatic peripheral arterial disease or carotid stenosis

**DOI:** 10.1186/s12872-026-06568-0

**Published:** 2026-09-16

**Authors:** Sofia Skröder, Dritan Poci, Daniel Robert Smith, Mats Dreifaldt, Allan Sirsjö, Espen Fengsrud, Anna Björkenheim, Liza Ljungberg

**Affiliations:** 1 School of Medical Sciences, Faculty of Medicine and Health, Örebro University, Örebro, Sweden; 2 Centre for Clinical Research and Education, Region Värmland, Karlstad, Sweden; 3 Department of Clinical Physiology, Sahlgrenska University Hospital, Gothenburg, Sweden; 4 Department of Molecular and Clinical Medicine, Institute of Medicine, Sahlgrenska Academy, University of Gothenburg, Gothenburg, Sweden; 5 Clinical Epidemiology and Biostatistics, Faculty of Medicine and Health, School of Medical Sciences, Örebro University, Örebro, 70182 Sweden; 6 Institute of Cancer Research, London, UK; 7 Department of Cardiothoracic Surgery, Örebro University Hospital and University Health Care Research Centre, Örebro, 70182 Sweden; 8 Department of Cardiology, Faculty of Medicine and Health, Örebro University, Örebro, 70182 Sweden

**Keywords:** Atrial fibrillation, Atherosclerosis, Biomarker, Bone morphogenetic proteins, Cardiovascular disease

## Abstract

**Background:**

Bone morphogenetic protein 10 (BMP10) has atrial-specific and vascular properties and may reflect both atrial and vascular pathophysiology. We investigated whether BMP10 levels were associated with new-onset atrial fibrillation (AF), major adverse cardiovascular events (MACE), and all-cause mortality after open revascularisation for symptomatic peripheral arterial disease (PAD) or carotid stenosis (CS).

**Methods:**

Patients scheduled for endarterectomy for symptomatic PAD or CS were enrolled. BMP10 concentrations were measured using a multiplex immunoassay. Clinical outcomes were obtained from medical records during one-year follow-up. Associations between BMP10 and outcomes were assessed using Cox proportional hazards regression.

**Results:**

A total of 210 patients were included (63% men; 61% underwent surgery for PAD). Women were older than men and had higher BMP10 levels. Forty patients had known preoperative AF. During one-year follow-up, 26 patients experienced MACE and 13 died. Among 170 patients without AF at baseline, 10 developed new-onset AF.

In unadjusted Cox regression, higher BMP10 levels were associated with MACE (HR 1.01, 95% CI 1.00-1.03; *p* = 0.047) and all-cause mortality (HR 1.03, 95% CI 1.01–1.04; *p* < 0.001), but not with new-onset AF (HR 1.00, 95% CI 0.97–1.03; *p* = 0.80). Effect estimates were similar after adjustment for age and sex. Model-based point estimates of one-year cumulative incidence were numerically higher at higher BMP10 concentrations for MACE and all-cause mortality, although confidence intervals were wide; no corresponding pattern was observed for new-onset AF.

**Conclusions:**

In this exploratory cohort, higher BMP10 levels were associated with MACE and all-cause mortality after endarterectomy for symptomatic PAD or CS. Women had higher BMP10 levels than men. These findings should be interpreted cautiously and considered hypothesis-generating; larger studies are required to validate the observed associations.

## Introduction

Bone morphogenetic protein 10 (BMP10) is an atrial-enriched protein that is predominantly expressed in the right atrial myocardium in adults. During embryonic cardiac development, BMP10 plays an essential role in cardiac morphogenesis, including ventricular wall formation and the establishment of left-right asymmetry [1–3]. BMP10 has also been shown to influence endothelial activation, apoptosis, angiogenesis, vascular homeostasis and vascular contractility [1, 3]. It primarily signals through activin receptor-like kinase 1, which is largely expressed on vascular endothelial cells but varies across vascular beds, suggesting that BMP10 may exert heterogeneous effects in different vascular territories [2].

Murine and human models suggest that BMP10 also plays an important role in cardiovascular (CV) pathology [2–5]. Previous studies have reported associations between BMP10 and atherosclerotic disease, potentially through effects on monocyte recruitment to the vascular endothelium and pathways related to endothelial dysfunction and fibrosis [6]. BMP10 has also been associated with adverse clinical outcomes such as recurrent atrial fibrillation (AF) episodes and ischaemic stroke in patients with established AF [7–14]. In contrast to its beneficial role during embryonic cardiac and vascular development [1–3], elevated circulating BMP10 levels in adults have been associated with atrial stress and remodelling [4, 5]. Given its atrial specificity and vascular effects, BMP10 may be particularly relevant in patients with atherosclerotic disease, with or without AF [15].

Sex-related differences in circulating BMP10 concentrations have also been described, with higher levels in women than in men in the general population [16], among patients with AF [12–14], and in cohorts undergoing AF ablation [17].

Patients with established atherosclerotic CV disease constitute a high-risk population with a substantial burden of recurrent CV events [18]. Atherosclerosis commonly affects the coronary, peripheral, and cerebrovascular arterial beds and is a major contributor to myocardial infarction, ischaemic stroke, and death. Peripheral arterial disease (PAD) and carotid stenosis (CS) represent clinically distinct manifestations of atherosclerotic disease. Revascularisation in PAD is primarily performed to relieve symptoms and preserve limb function, whereas intervention for CS aims to prevent ischaemic stroke [19, 20]. Despite these differences, both conditions are associated with a high risk of AF and future CV events.

Circulating biomarkers are of increasing clinical interest and may contribute to risk assessment in patients with CV disease. However, the association between BMP10 levels and atherosclerotic disease in high‑risk vascular populations remains unclear. Sex-related differences in BMP10 levels are also insufficiently characterised in patients with advanced atherosclerotic disease.

This study aimed to evaluate the associations between circulating BMP10 and new-onset AF, major adverse CV events (MACE), and all-cause mortality within one year after open revascularisation in patients with symptomatic PAD or CS, and to further examine potential sex differences in BMP10 levels in this population.

## Patients and methods

### Study population and data collection

This exploratory cohort study comprises patients enrolled in the ongoing multicentre *Atherosclerosis – Lipids and Inflammation in Peripheral Arterial Disease and Carotid Arteries* (ALIPACA) trial. Participants undergoing endarterectomy for symptomatic PAD or CS were recruited at three sites in mid-Sweden: Karlstad Central Hospital, Västerås Central Hospital, and Örebro University Hospital.

The ALIPACA inclusion criteria were age ≥ 18 years, ability to provide written informed consent, and eligibility for endarterectomy. Ongoing immunosuppressive treatment was an exclusion criterion. Patients enrolled between 2019 and 2024 were eligible for the present substudy.

Because recruitment to the underlying ALIPACA cohort was ongoing, the present substudy was limited to participants who had completed one-year follow-up and had available plasma samples and accessible clinical records. Patients for whom relevant clinical records could not be retrieved from the hospitals’ medical record systems were not included in the analyses. The final study sample comprised 210 participants.

Written informed consent was obtained from all study participants prior to any study-related procedures. No formal a priori sample-size or power calculation was performed because this was an exploratory analysis within an ongoing cohort. The available sample size was determined by the number of eligible participants with available plasma aliquots for biomarker measurements and accessible one-year follow-up data.

Venous blood samples were collected on the day of surgery immediately before anaesthesia. All participants underwent a clinical preoperative assessment and completed a questionnaire covering demographics, smoking status, comorbidities, and medication use. Baseline clinical variables and one-year follow-up data on CV risk factors, AF status, MACE, and mortality were retrieved from each hospital’s medical record system.

MACE was defined as a composite of non-fatal ischaemic stroke, non-fatal myocardial infarction, and all-cause mortality. Time-to-event analyses were based on the time from surgery to the first MACE during follow-up, if multiple, during the follow-up period. A prespecified three-point MACE definition was applied, using all-cause mortality rather than CV mortality because cause-of-death adjudication is frequently uncertain in older patients with multiple comorbidities. Furthermore, we could not guarantee the accuracy of the data on CV mortality. Use of all‑cause mortality as the death component in three‑point MACE is well established in observational CV research, where cause‑specific mortality is often unavailable or unreliable, and systematic reviews have shown that all‑cause death is more commonly used than CV death in MACE composites [21, 22].

Preoperative AF was defined as a physician-established diagnosis of paroxysmal, persistent, permanent AF, or unspecified AF documented before endarterectomy and identified using ICD-10-SE codes I48.0, I48.1, I48.2, and I48.9. New-onset AF was defined as the first physician-established and electrocardiographically confirmed AF diagnose recorded in the medical record within one year after endarterectomy in a patient without documented preoperative AF.

Among patients undergoing surgery for CS, ischaemic stroke occurring within 30 days postoperatively was classified as a perioperative event and was not included in the one-year MACE or new non-fatal ischaemic stroke endpoints. Ischaemic stroke occurring more than 30 days after surgery was classified as a new event, in accordance with the commonly applied 30-day perioperative risk timeframe described in European guidelines [23]. Baseline ischaemic stroke was defined based on self-reported prior stroke and/or documented medical history.

### Blood sampling and preparation

Blood samples were collected in EDTA tubes (K2 EDTA, BD Vacutainer, Franklin Lakes, NJ, USA), centrifuged at room temperature (22 °C) for 10 min at 2000 g, aliquoted, and stored at -80 °C until analysis, in accordance with national principles for healthcare-integrated biobanking [24].

### Luminex analysis

BMP10 concentrations were measured using the Luminex Human Discovery Assay (R&D Systems, Minneapolis, MN, USA). Plasma samples were randomised across six plates according to sex, study site, smoking status, and surgical indication.

Assays were performed according to the manufacturer’s instructions, and all samples were analysed in duplicate. Pooled plasma from nine participants was included on each plate as a plate control and was used for normalisation. Intra-plate variability was 3.0%, calculated as the coefficient of variation of duplicate measurements. Inter-plate variability was 14.1%, calculated as the coefficient of variation of the six plate-control samples. To account for between-plate variation, normalised concentrations were used in all statistical analyses. Normalisation was performed by dividing each sample’s raw concentration by the plate-specific control value, which reduces systematic plate-to-plate differences and is standard practice in bead-based multiplex assays. Values below the lowest standard concentration were imputed as one-half of the lowest standard concentration.

### Statistical analysis

Descriptive statistics were used to summarise demographic and clinical characteristics. Continuous variables were assessed for normality using the Shapiro-Wilk test and are presented as mean (standard deviation) or median (interquartile range, IQR), as appropriate. Correlations were evaluated using Spearman correlation or Pearson point-biserial correlation, as appropriate. Comparisons among three or more groups were performed using analysis of variance (ANOVA) or the Kruskal-Wallis test, depending on data distribution. For subgroup and post-hoc comparisons, the unpaired t-test was used for normally distributed variables and the Mann-Whitney U test for skewed variables. Categorical variables were compared using Pearson’s chi-square test or Fisher’s exact test, as appropriate. Statistical significance was defined as a two-sided *p*-value < 0.05. Given the exploratory nature of the study, no adjustment was made for multiple testing. All *p*-values are therefore nominal and should be interpreted cautiously.

Time-to-event analyses for all outcomes were performed using crude and adjusted Cox proportional hazards regression models. BMP10 was included as a continuous linear predictor to preserve model stability and limit model complexity given the small number of outcome events. More flexible functional forms, including spline modelling, were not applied because the additional degrees of freedom would have resulted in unstable estimates, and formal tests of non-linearity would have had very low power in this sparse‑event setting. For each outcome, three models were fitted: a crude model, an age-adjusted model, and a model adjusted for age and sex. Hazard ratios with 95% confidence intervals were reported and represent the change in hazard per 1-pg/mL increase in BMP10.

To facilitate interpretation, model-based contrasts were evaluated at representative BMP10 concentrations corresponding to the empirical quartile values of the observed distribution: Q1 (22 pg/mL), Q2 (median; 38 pg/mL), and Q3 (52 pg/mL). From the fitted Cox models, contrasts comparing Q3 versus Q2 and Q3 versus Q1 were obtained. For each outcome, we calculated (i) one-year risk ratios, (ii) one-year cumulative incidence estimates, and (iii) absolute differences in one-year cumulative incidence. One-year cumulative incidence was derived from the fitted Cox models. Confidence intervals for all model-based contrasts were estimated using non-parametric bootstrap resampling with 300 replicates. The number of bootstrap replications was chosen to balance computational feasibility with the limited number of events, which restricts the precision achievable through resampling. Contrasts were conditional, with covariates fixed at typical values, defined as the median for continuous variables and mode for categorical variables. The proportional hazards assumption was assessed using Schoenfeld residuals, implemented using the cox.zph() function in R. Neither the global tests nor the covariate-specific tests provided meaningful evidence of violation of the proportional hazards assumption.

Kaplan-Meier curves were generated to visualise unadjusted differences in event-free survival across BMP10 tertiles, and differences were assessed using the log-rank test.

All analyses were performed using SPSS version 30 (IBM, Armonk, NY, USA) and R version 4.4.0 (R Foundation for Statistical Computing, Vienna, Austria).

## Results

### Study population and baseline characteristics

Of 234 potentially eligible participants, 210 were included in the present analysis. Twenty-four participants were excluded because their clinical records could not be accessed. Demographics and baseline characteristics are presented in Table 1. The median age was 76 years (IQR 9). The cohort was predominantly male (63%), and the surgical indication was more often symptomatic PAD than CS (61% versus 39%). At baseline, 40 patients (19%) had known AF. Women were older than men (*p* = 0.046), had a higher prevalence of hypertension (*p* = 0.009), a lower prevalence of diabetes (*p* = 0.041), and higher circulating BMP10 levels (*p* = 0.045). There was no correlation between BMP10 levels and age.

**Table 1:** Baseline characteristics

	Total (*n* = 210)	Men (*n* = 133)	Women (*n* = 77)	*p*
Indication for surgery: PAD, *n* (%)	128 (61.0)	70 (52.6)	58 (75.3)	**0.001**
Age at surgery (years), median (IQR)	76 (9)	75 (10)	77 (7)	**0.046**
BMP10 (pg/mL), median (IQR)	37.8 (30.1)	37.2 (33.1)	39.9 (26.9)	**0.045**
Heart failure, *n* (%)	33 (15.7)	22 (16.5)	11 (14.3)	0.665
Hypertension, *n* (%)	194 (92.4)	118 (88.7)	76 (98.7)	**0.009**
Diabetes, *n* (%)	76 (36.2)	55 (41.4)	21 (27.3)	**0.041**
Stroke/TIA/systemic embolism, *n* (%)	84 (40.0)	58 (43.6)	26 (33.8)	0.161
CHA₂DS₂-VA, median (IQR)	5 (2)	5 (2)	4 (2)	0.409
AF at baseline, *n* (%)	40 (19.0)	25 (18.8)	15 (19.5)	0.903

Baseline characteristics of included study participants (*n* = 210). Statistically significant differences (*p* < 0.05) are shown in bold

*Abbreviations*: *AF* atrial fibrillation, *BMP10* bone morphogenetic protein 10, *CHA₂DS₂-VA* congestive heart failure, hypertension, age ≥ 75 years (2 points), diabetes mellitus, stroke/TIA/systemic embolism (2 points), vascular disease, age 65–74 years, *IQR* interquartile range, *PAD* peripheral arterial disease, *TIA* transient ischaemic attack

All characteristics stratified by indication for surgery are presented in Table 2.

**Table 2:** Baseline characteristics stratified by surgical indication

	PAD (*n* = 128)	CS (*n* = 82)	*p*
Male sex, *n* (%)	70 (54.7)	63 (76.8)	**0.001**
Age at surgery (years), median (IQR)	76.0 (7.0)	74.0 (10.0)	**0.013**
BMP10 (pg/mL), median (IQR)	39.7 (28.2)	32.8 (27.7)	**0.033**
Heart failure, *n* (%)	28 (21.9)	5 (6.1)	**0.002**
Hypertension, *n* (%)	120 (93.8)	74 (90.2)	0.350
Diabetes, *n* (%)	56 (43.8)	20 (24.4)	**0.004**
Stroke/TIA/systemic embolism, *n* (%)	21 (16.4)	63 (76.8)	**< 0.001**
CHA2DS2-VA, median (IQR)	4.0 (1.0)	5.0 (2.0)	**0.002**
AF at baseline, *n* (%)	27 (21.1)	13 (15.9)	0.345

Baseline characteristics of included study participants (*n* = 210). Statistically significant differences (*p* < 0.05) are shown in bold

*Abbreviations*: *AF* atrial fibrillation, *BMP10* bone morphogenetic protein 10, *CHA₂DS₂-VA* congestive heart failure, hypertension, age ≥ 75 years (2 points), diabetes mellitus, stroke/TIA/systemic embolism (2 points), vascular disease, age 65–74 years, *CS* carotid stenosis, *IQR* interquartile range, *PAD* peripheral arterial disease, *TIA* transient ischaemic attack

### Clinical outcomes within one-year follow-up

Among patients without preoperative AF (*n* = 170), 10 (5.9%) were diagnosed with new-onset AF during the first year after endarterectomy. The remaining 160 (94.1%) participants had no documented AF during follow-up. A total of 26 participants experienced at least one MACE. Individual MACE components were not mutually exclusive. Thirteen patients died.

No significant sex differences were observed for new-onset AF, one-year non-fatal ischaemic stroke, non-fatal acute myocardial infarction, MACE, or all-cause mortality (Table 3). No significant differences in one-year clinical outcomes were observed between surgical indication groups (Table 4).

**Table 3:** Clinical outcomes within one-year follow-up

	Total (*n* = 210)	Men (*n* = 133)	Women (*n* = 77)	*p*
New non-fatal ischaemic stroke at 1 year, *n* (%)	10 (4.8)	7 (5.3)	3 (3.9)	0.696
New non-fatal AMI at 1 year, *n* (%)	7 (3.3)	5 (3.8)	2 (2.6)	0.694
All-cause mortality at 1 year, *n* (%)	13 (6.2)	8 (6.0)	5 (6.5)	0.890
MACE^a^ at 1 year, *n* (%)	26 (12.4)	17 (12.8)	9 (11.7)	0.817
New-onset AF^b^ at 1 year, *n* (%)	10 (5.9)	7 (6.5)	3 (4.8)	0.748

Clinical outcomes within one-year follow-up after open revascularisation in symptomatic peripheral arterial disease or carotid stenosis (*n* = 210). Perioperative ischaemic strokes occurring within 30 days after carotid endarterectomy were not included in the one-year MACE or new non-fatal ischaemic stroke endpoint

*Abbreviations*: *AF* atrial fibrillation, *AMI* acute myocardial infarction, *MACE* major adverse cardiovascular events

^a^ MACE was defined as a composite outcome of non-fatal ischaemic stroke, non-fatal AMI, and all-cause mortality

^b^ New-onset AF among participants without AF at baseline (*n*_*total*_=170, *n*_men_=108, and *n*_women_=62)

**Table 4:** Clinical outcomes within one-year follow-up stratified by surgical indication

	PAD (*n* = 128)	CS (*n* = 82)	*p*
New non-fatal ischaemic stroke at 1 year, *n* (%)	4 (3.1)	6 (7.3)	0.257
New non-fatal AMI at 1 year, *n* (%)	4 (3.1)	3 (3.7)	1.000
All-cause mortality at 1 year, *n* (%)	9 (7.0)	4 (4.9)	0.528
MACE^a^ at 1 year, *n* (%)	15 (11.7)	11 (13.4)	0.716
New-onset AF ^b^ at 1 year, *n* (%)	5 (5.0)	5 (7.2)	0.529

Clinical outcomes within one-year follow-up after open revascularisation in symptomatic PAD or CS, stratified by surgical indication (*n* = 210). Perioperative ischaemic strokes occurring within 30 days after carotid endarterectomy were not included in the one-year MACE or new non-fatal ischaemic stroke endpoint

*Abbreviations*: *AF* atrial fibrillation, *AMI* acute myocardial infarction, *CS* carotid stenosis, *MACE* major adverse cardiovascular events, *PAD* peripheral arterial disease

^a^ MACE was defined as a composite outcome of non-fatal ischaemic stroke, non-fatal AMI, and all-cause mortality

^b^ New-onset AF among participants without AF at baseline (*n*_*total*_=170, *n*_PAD_=101, and *n*_CS_=69)

### Associations between BMP10 and clinical outcomes

**Fig. 1:**
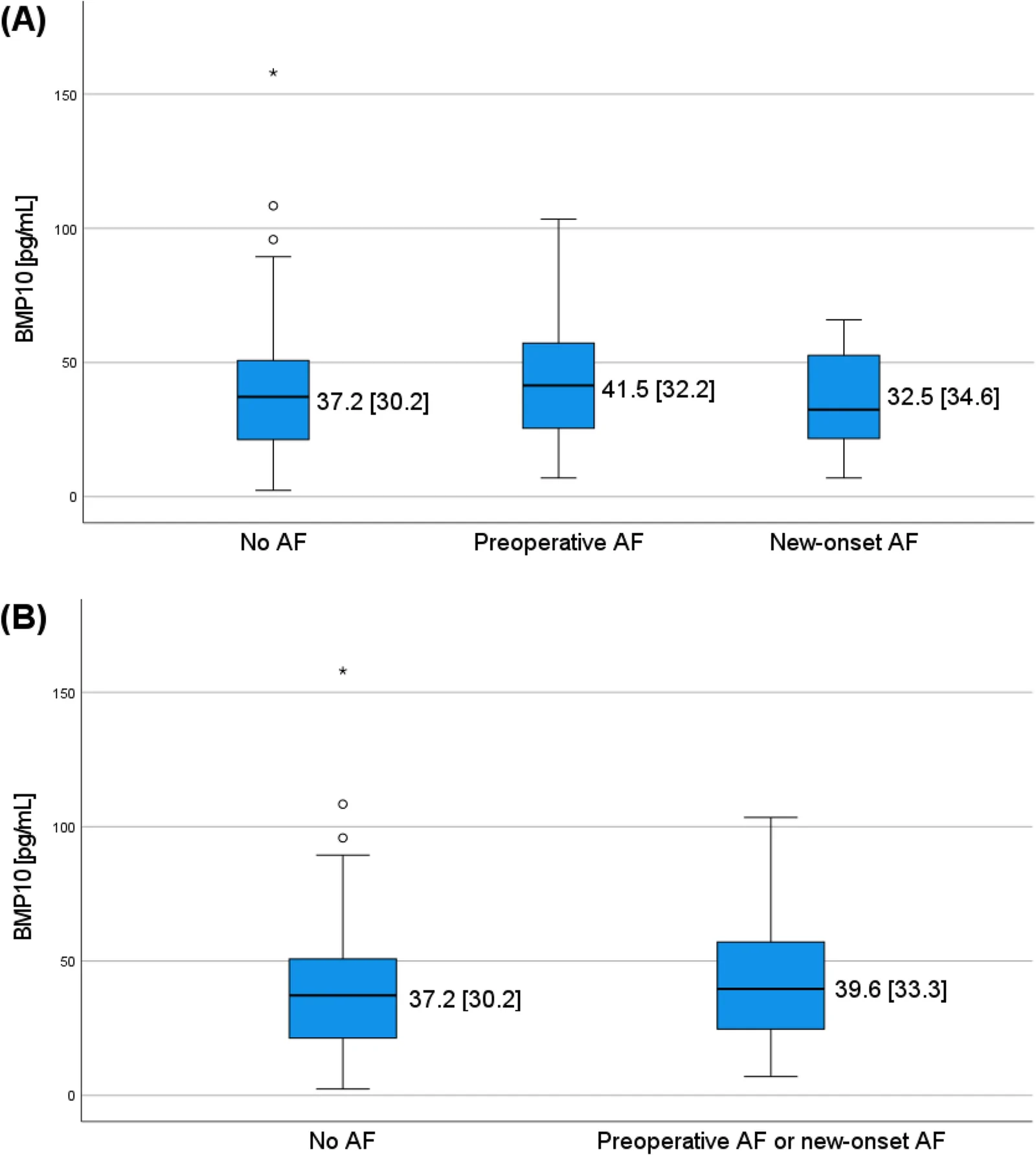
BMP10 levels according to AF status. **A** BMP10 levels in patients with no AF (*n*=160), preoperative AF (*n*=40), new-onset AF (*n*=10). **B** BMP10 levels in patients with no AF (*n*=160) compared with patients with either preoperative AF or new-onset AF (*n*=50). Values are shown as median [IQR]. Open circles indicate mild outliers and asterisks indicate extreme outliers, according to the software-defined boxplot criteria. *Abbreviations*: AF, atrial fibrillation; BMP10, bone morphogenetic protein 10; IQR, interquartile range

BMP10 levels did not differ among patients with preoperative AF, those with new-onset AF during follow-up, and those without AF (Fig. 1A). Similarly, BMP10 levels did not differ when comparing patients with either preoperative or new-onset AF with those who had no documented AF throughout follow-up (Fig. 1B).

**Fig. 2:**
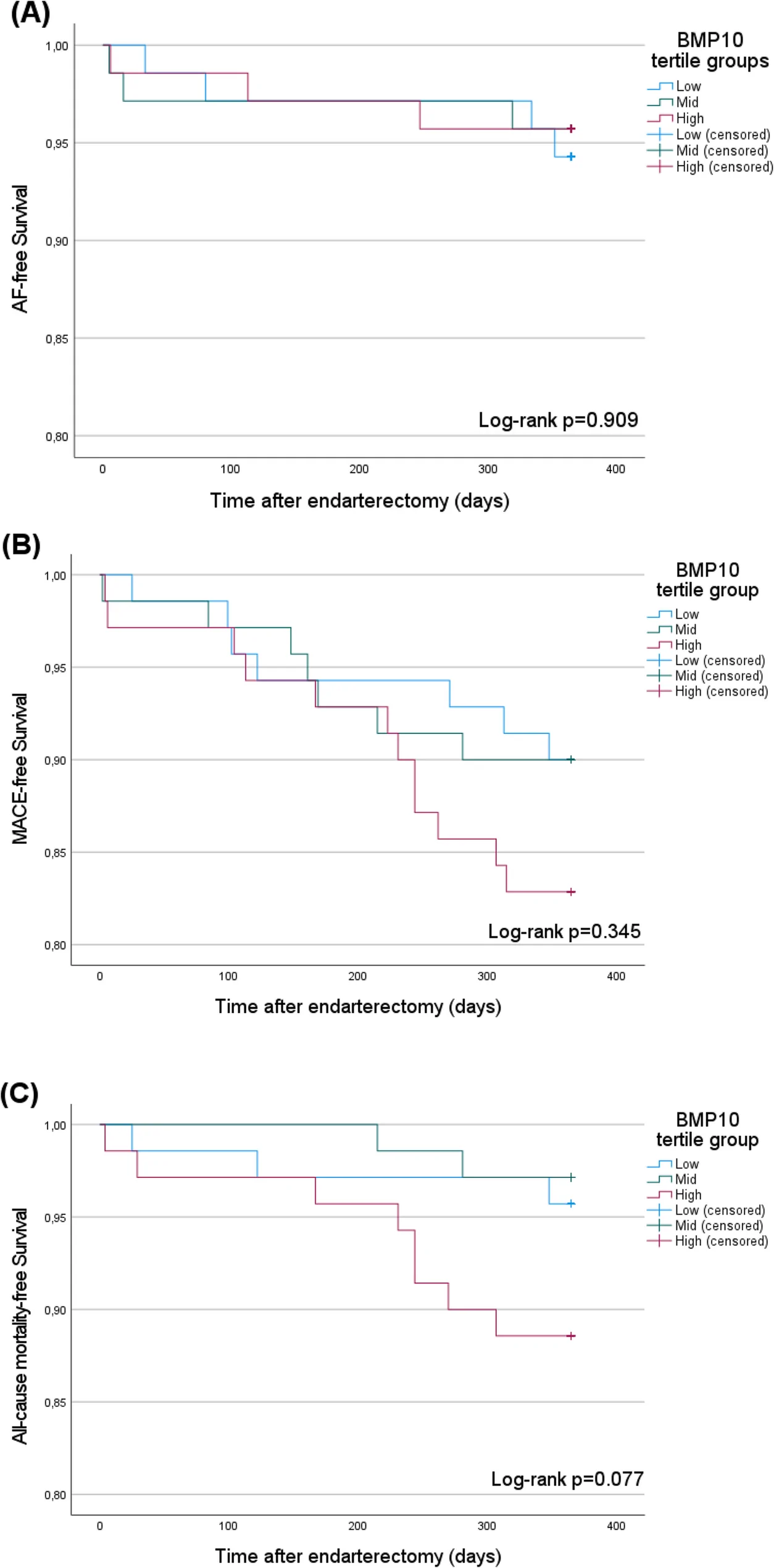
Kaplan-Meier analyses by BMP10 tertiles. Kaplan-Meier curves showing event-free survival across tertiles of BMP10 for (**A**) new-onset AF, (**B**) MACE, and (**C**) all-cause mortality within one year after endarter-ectomy (*n*=210). Panel **A** includes participants without AF at baseline (*n*=170); panels **B** and **C** include the full cohort (*n*=210). *Abbreviations*: AF, atrial fibrillation; BMP10, bone morphogenetic protein 10; MACE, major adverse cardiovascular events

Kaplan-Meier time-to-event analyses were performed to describe associations between BMP10 levels and the occurrence of new-onset AF, MACE, and all-cause mortality within one year after surgery. Ten patients developed new-onset AF during follow-up: four in the lowest BMP10 tertile, three in the middle tertile, and three in the highest tertile. Kaplan-Meier curves showed no differences in AF-free survival across tertiles (*p* = 0.909) (Fig. 2A).

A total of 26 MACE occurred within one year postoperatively, with seven events in each of the two lower BMP10 tertiles and 12 events in the highest tertile. There was no significant difference in MACE-free survival across tertiles (*p* = 0.345) (Fig. 2B).

During follow-up, 13 deaths occurred: three in the lowest BMP10 tertile, two in the middle tertile, and eight in the highest tertile. Estimated postoperative survival did not differ significantly across BMP10 tertiles (*p* = 0.077) (Fig. 2C).

In crude Cox proportional hazards models, higher BMP10 levels were associated with a higher hazard of MACE (HR: 1.01, 95% CI: 1.00-1.03, *p* = 0.047) and all-cause mortality (HR: 1.03, 95% CI: 1.01–1.04, *p* < 0.001). No statistically significant association was detected with new-onset AF (HR: 1.00, 95% CI: 0.97–1.03, *p* = 0.80), although this analysis was limited by only 10 events. Effect estimates were similar after adjustment for age and sex (Table 5).

**Table 5:** Hazard ratios for clinical outcomes across crude and adjusted models

	Crude HR (9% CI)	*p*	Age-adjusted HR (95% CI)	*p*	Age- and sex-adjusted HR (95% CI)	*p*
New-onset AF	1.00 (0.97–1.03)	0.80	0.99 (0.97–1.02)	0.70	1.00 (0.97–1.03)	0.80
MACE	1.01 (1.00-1.03)	**0.047**	1.02 (1.00-1.03)	**0.049**	1.02 (1.00-1.03)	**0.045**
ACM	1.03 (1.01–1.04)	**< 0.001**	1.03 (1.01–1.05)	**< 0.001**	1.03 (1.01–1.05)	**< 0.001**

Hazard ratios are reported per 1-pg/mL increase in BMP10, with 95% confidence intervals and *p*-values for the associations with new-onset AF, MACE, and all-cause mortality within one year after endarterectomy in patients with peripheral arterial disease or carotid stenosis. Estimates are shown for crude, age-adjusted, and age- and sex-adjusted Cox proportional hazards models. Significant associations are indicated in bold (*p* < 0.05). Perioperative ischaemic strokes occurring within 30 days after carotid endarterectomy were not included in the one-year MACE endpoint.

*Abbreviations*: *ACM* all-cause mortality, *AF* atrial fibrillation, *CI* confidence interval, *HR* hazard ratio, *MACE* major adverse cardiovascular events

**Fig. 3:**
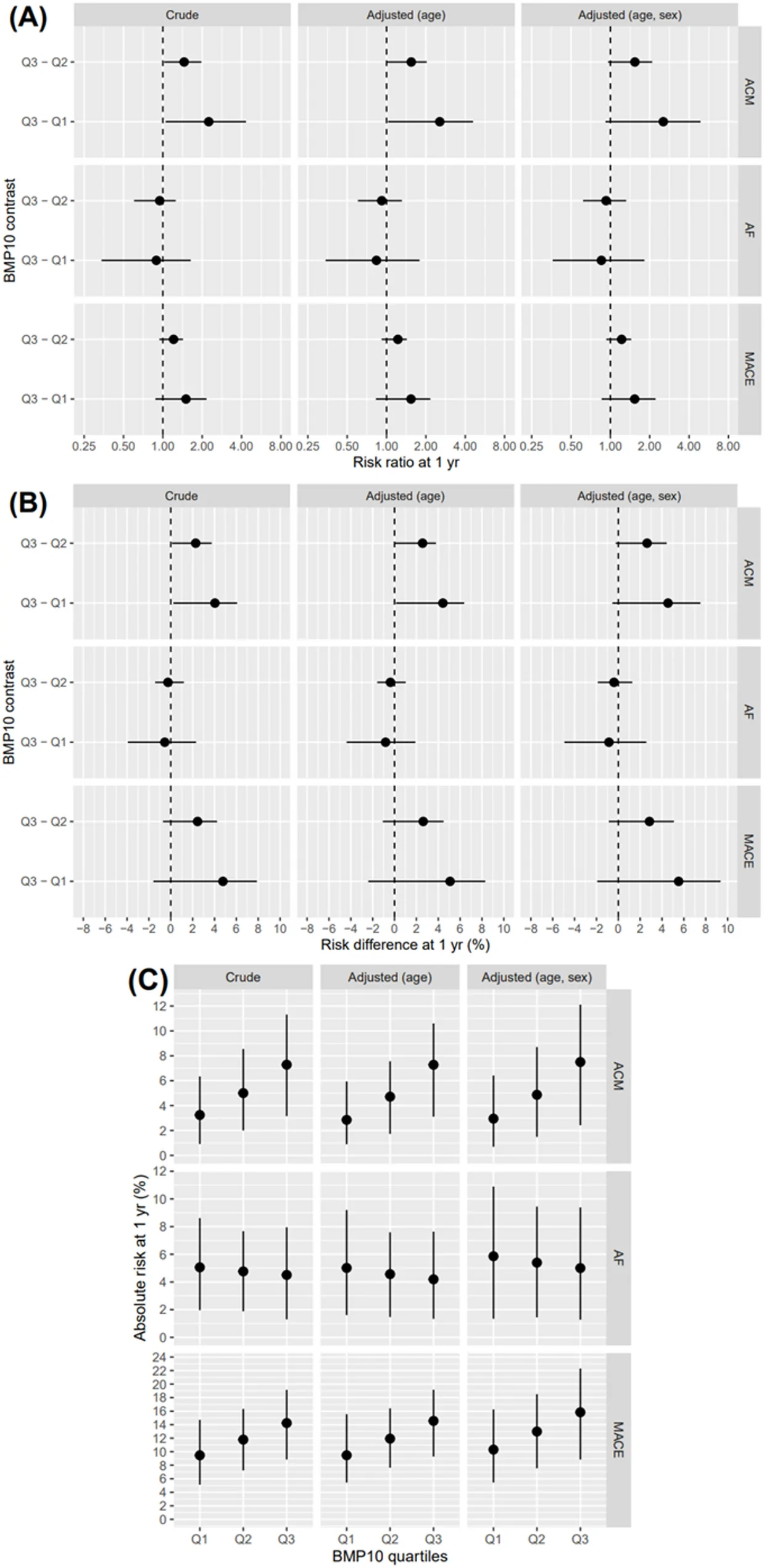
Model-based associations between BMP10 levels and clinical outcomes in patients undergoing endarterectomy for symptomatic peripheral arterial disease or carotid stenosis.** A** Model-based one-year risk ratios for new-onset AF, MACE, and all-cause mortality comparing BMP10 at Q3 with Q2 and Q1, derived from crude and adjusted Cox proportional hazards models. **B** Absolute differences in model-based one-year cumulative incidence for new-onset AF, MACE, and all-cause mortality comparing Q3 with Q2 and Q1. **C** Model-based one-year cumulative incidence of new-onset AF, MACE, and all-cause mortality at BMP10 concentrations corresponding to Q1, Q2, and Q3. Q1, Q2, and Q3 correspond to BMP10 concentrations of 22, 38, and 52 pg/mL, respectively. Models are shown without adjustment, with adjustment for age, and with adjustment for age and sex. Covariates were fixed at the median for continuous variables and mode for categorical variables. Error bars indicate 95% confidence intervals based on 300 bootstrap replications. Perioperative ischaemic strokes occurring within 30 days after carotid endarterectomy were not included in the one-year MACE endpoint. *Abbreviations*: ACM, all-cause mortality; AF, atrial fibrillation; BMP10, bone morphogenetic protein 10; MACE, major adverse cardiovascular events

Model-based relative contrasts comparing Q3 with Q2 and Q1 showed numerically higher one-year risk estimates for MACE and all-cause mortality, whereas no consistent pattern was observed for new-onset AF (Fig. 3A). Model-based point estimates of one-year cumulative incidence were likewise numerically higher at Q3 than at Q2 and Q1 for MACE and all-cause mortality, although confidence intervals were wide. No consistent corresponding pattern was observed for new-onset AF.

Absolute differences in one-year cumulative incidence were similar after covariate adjustment (Fig. 3B), and model-based one-year cumulative incidence at Q1, Q2, and Q3 is presented in Fig. 3C.

## Discussion

In this exploratory cohort of patients undergoing open vascular surgery for symptomatic PAD or CS, higher circulating BMP10 levels were associated with a higher one-year risk of MACE and all-cause mortality. No statistically significant association was detected with new-onset AF, and BMP10 levels did not differ when patients with preoperative or new-onset AF were considered together. However, the analysis of new-onset AF was underpowered because only 10 events occurred. Effect estimates were similar after adjustment for age and sex. Model-based point estimates of one-year cumulative incidence were numerically higher at higher BMP10 concentrations for MACE and all-cause mortality, although confidence intervals were wide. Women had higher circulating BMP10 levels than men, consistent with findings from previous studies in other populations [14, 16, 17].

Circulating BMP10 has primarily been evaluated in populations with established AF, in which higher levels have been associated with AF recurrence, MACE, stroke, and mortality [12–14]. The present study extends the evaluation of BMP10 to a population undergoing open vascular surgery for advanced atherosclerotic disease. In this setting, higher BMP10 levels were associated with MACE and all‑cause mortality, whereas no statistically significant association was detected with new‑onset AF. These findings may indicate that BMP10 reflects a broader burden of atrial, vascular, or systemic CV disease rather than a specific propensity to develop clinically recognised AF. However, given the observational design, mechanistic or causal conclusions cannot be drawn.

All-cause mortality was used as the death component of the composite MACE endpoint because reliable adjudication of cause-specific mortality was not available. Although this avoids uncertain classification of cause of death, it reduces the CV specificity of the composite endpoint, and non-CV deaths may therefore have contributed to the observed associations with MACE. The individual components of the composite endpoint are presented separately in Tables 3 and 4, allowing assessment of their contribution across sex and surgical indication. Detailed and sufficiently standardised data on procedural success, reinterventions, and perioperative complications other than perioperative stroke were not available for all participants and were therefore not included in the analysis. Consequently, we could not determine whether procedural factors contributed to postoperative MACE or whether higher baseline BMP10 levels partly reflected more advanced disease associated with greater procedural risk.

Kaplan-Meier analyses did not demonstrate statistically significant differences across BMP10 tertiles, although the event curves suggested numerically higher one-year rates of MACE and mortality in the highest tertile. In contrast, Cox regression models using BMP10 as a continuous predictor indicated associations with MACE and all-cause mortality. The two approaches use different parameterisations: the Kaplan-Meier analyses categorised BMP10 into tertiles, whereas the Cox models retained BMP10 as a continuous linear predictor. Categorisation may reduce statistical power by discarding information on variation within groups, and the two methods therefore test different aspects of the associations. Because the discrepancy was also present in the crude Cox model, it cannot be explained by covariate adjustment alone. These findings should therefore be interpreted cautiously and not as evidence of a robust difference between the analytical approaches.

The proportional hazards assumption was evaluated using Schoenfeld residuals, and neither the global nor covariate-specific tests provided meaningful evidence of violation. However, these tests had limited ability to detect anything other than marked departures from proportional hazards, and non-significant results should not be interpreted as proof that the assumption was fully satisfied. Confidence intervals for the model-based contrasts were estimated using 300 bootstrap replications and should therefore be regarded as approximate. Their precision is additionally constrained by the limited amount of event information in the dataset.

Biomarker-outcome relationships may be nonlinear, and the broad dispersion of BMP10 values in this cohort raises the possibility of threshold effects or influence from extreme observations. However, the small number of events precluded reliable spline‑based or other flexible modelling approaches, which would have required additional degrees of freedom and resulted in unstable estimates. BMP10 was therefore modelled as a continuous linear predictor to limit model complexity. Formal testing of nonlinearity was not performed, and potential nonlinear associations cannot be excluded. Extreme BMP10 observations were retained because there was no evidence that they represented measurement or data-quality errors and no exclusion threshold had been prespecified. The estimates may nevertheless be sensitive to individual observations. Larger studies with sufficient event counts will be required to evaluate potential nonlinear associations and the influence of extreme biomarker values more reliably.

Twenty-four potentially eligible participants were excluded because their clinical records could not be accessed. As no baseline or outcome data were available for these individuals, differences between included and excluded participants could not be assessed. Selection bias therefore cannot be excluded, and the direction and magnitude of any such bias cannot be determined.

The study population combined patients with symptomatic PAD and CS, which are clinically and pathophysiologically distinct manifestations of atherosclerotic disease. They differ in symptom presentation, indications for surgery, expected postoperative complications, and mechanisms of recurrent CV events. For example, recurrent ischaemic stroke after carotid endarterectomy is mechanistically distinct from limb-related ischaemic complications following peripheral vascular surgery [20, 25]. Although baseline characteristics and clinical outcomes were presented separately by surgical indication, the limited number of events did not support reliable subgroup Cox models or formal interaction testing. Pooling was therefore a pragmatic choice for this exploratory analysis and should not be interpreted as evidence that the associations between BMP10 and outcomes were identical across vascular territories. The pooled estimates should be interpreted in the context of this clinical heterogeneity.

Recruitment to the underlying ALIPACA cohort was ongoing, and the present substudy was limited to participants who had completed one-year follow-up and had available plasma samples and accessible clinical records, resulting in a final sample of 210 patients. No formal a priori sample-size or power calculation was performed. The small number of outcome events limited statistical precision, reduced the ability to detect modest associations, and restricted the extent of multivariable adjustment.

Preoperative and new-onset AF were defined by physician-established clinical diagnoses supported by electrocardiographic documentation in the medical records. However, systematic or prolonged rhythm monitoring was not performed, and asymptomatic or paroxysmal AF may therefore have remained undetected. However, this may have resulted in underestimation of the incidence of new-onset AF and reduced the ability to detect an association with BMP10 [26]. Information on heart rhythm at the time of blood sampling was also unavailable and may have influenced circulating BMP10 levels [4]. Given that only 10 new-onset AF events occurred, the AF analysis was underpowered, and the absence of a statistically significant association should be interpreted as inconclusive rather than as evidence of no association. Prospective studies with systematic rhythm monitoring and larger numbers of AF events are required.

Several outcomes and model specifications were evaluated without adjustment for multiple testing. The reported *p*-values are consequently nominal and should not be interpreted as confirmatory evidence. This is particularly relevant to the association between BMP10 and MACE, for which the *p*-values were close to the conventional threshold for statistical significance and the confidence intervals approached the null. The association may therefore be sensitive to small changes in the data or model specification and should be regarded as exploratory and hypothesis-generating. Both type I and type II errors remain possible.

Given the limited information available relative to the number of potential confounders, only a restricted set of covariates could be included in the adjusted models to minimise the risk of overfitting. The adjusted models were deliberately parsimonious and included age and sex. Including additional clinically relevant comorbidities such as heart failure, diabetes, hypertension, or renal dysfunction would have increased the risk of overfitting and produced unstable estimates. Residual confounding is therefore likely, and the observed associations cannot be interpreted as independent prognostic effects of BMP10. The number of events was also insufficient to evaluate discrimination, calibration, or reclassification. The study was not designed to develop or validate a prediction model, and the findings should not be interpreted as evidence that BMP10 provides incremental predictive or clinical utility beyond established CV risk factors.

Further investigation of potential sex differences in BMP10 levels and their associations with clinical outcomes is warranted. In our cohort, women had higher circulating BMP10 levels than men. Prior studies have reported sex-related variation in circulating BMP10 concentrations across CV populations, including patients with AF and atherosclerotic disease [14, 16, 17]. Women with AF have also been reported to have a higher symptom burden, more comorbidities, and more advanced CV disease at the time of treatment consideration than men [27]. Thus, the sex difference in BMP10 observed in our study may reflect differences in atrial, vascular, or systemic disease expression, although the cross-sectional nature of this comparison precludes causal interpretation. Hypertension is a recognised contributor to atrial and vascular stress, but its near‑universal prevalence in this cohort limited our ability to assess whether it contributed to the observed sex difference in baseline BMP10 levels. Further studies are needed to determine the mechanisms and potential clinical relevance of sex-related differences in circulating BMP10.

In conclusion, higher BMP10 levels were associated with MACE and all‑cause mortality in this exploratory vascular surgery cohort, whereas the findings for new-onset AF were inconclusive. The prognostic role of BMP10 remains uncertain, and the present findings should be considered hypothesis-generating. Larger, adequately powered studies with systematic rhythm monitoring, comprehensive assessment of potential confounders, adjudicated CV endpoints, and external validation are required to determine the clinical relevance of BMP10 in this setting.

## Data Availability

The research data include confidential patient information and will not be made publicly available. Datasets generated and/or analysed during the current study are available from the corresponding author upon reasonable request.

## Glossary

AF: Atrial fibrillation
ALIPACA: The Atherosclerosis – Lipids and Inflammation in Peripheral Arterial Disease and Carotid Arteries trial
BMP10: Bone morphogenetic protein 10
CHA₂DS₂-VA: Congestive heart failure, hypertension, age ≥75 years (2 points), diabetes mellitus, stroke/TIA/systemic embolism (2 points), vascular disease, age 65–74 years
CS: Carotid stenosis
CV: Cardiovascular
ICD: International Classification of Diseases
IQR: Interquartile range
MACE: Major adverse cardiovascular events
PAD: Peripheral arterial disease
TIA: Transient ischaemic attack

## References

[1] Wang X, Sun H, Yu H, Du B, Fan Q, Jia B, et al. Bone morphogenetic protein 10, a rising star in the field of diabetes and cardiovascular disease. J Cell Mol Med. 2024;28(10):e18324. 38760897 10.1111/jcmm.18324 PMC11101671

[2] Jiang H, Salmon RM, Upton PD, Wei Z, Lawera A, Davenport AP, et al. The Prodomain-bound Form of Bone Morphogenetic Protein 10 Is Biologically Active on Endothelial Cells. J Biol Chem. 2016;291(6):2954–66. 26631724 10.1074/jbc.M115.683292 PMC4742757

[3] Wang L, Rice M, Swist S, Kubin T, Wu F, Wang S, et al. BMP9 and BMP10 Act Directly on Vascular Smooth Muscle Cells for Generation and Maintenance of the Contractile State. Circulation. 2021;143(14):1394–410. 33334130 10.1161/CIRCULATIONAHA.120.047375

[4] Sommerfeld LC, Schrapers J, Muller KF, Bravo-Merodio L, Siebels B, Vermeer-Stoter AMS, et al. High Rate Triggers Increased Atrial Release of BMP10, A Biomarker for Atrial Fibrillation and Stroke, and BMP10 Affects Ventricular Cardiomyocytes. Circ Arrhythm Electrophysiol. 2025;18(11):e013834. 41090224 10.1161/CIRCEP.125.013834 PMC12629124

[5] Ceelen DCH, Bracun V, van Essen BJ, Voors AA, de Boer RA, Ter Maaten JM, et al. Circulating bone morphogenetic protein 10 as a novel marker of atrial stress and remodelling in heart failure. Heart. 2025;111(4):172–9. 39613454 10.1136/heartjnl-2024-324486

[6] Mitrofan CG, Appleby SL, Nash GB, Mallat Z, Chilvers ER, Upton PD, et al. Bone morphogenetic protein 9 (BMP9) and BMP10 enhance tumor necrosis factor-alpha-induced monocyte recruitment to the vascular endothelium mainly via activin receptor-like kinase 2. J Biol Chem. 2017;292(33):13714–26. 28646109 10.1074/jbc.M117.778506 PMC5566526

[7] Reyat JS, Chua W, Cardoso VR, Witten A, Kastner PM, Kabir SN et al. Reduced left atrial cardiomyocyte PITX2 and elevated circulating BMP10 predict atrial fibrillation after ablation. JCI Insight. 2020;5(16):e139179. 10.1172/jci.insight.139179 PMC7455124 32814717

[8] Winters J, Kawczynski MJ, Gilbers MD, Isaacs A, Zeemering S, Bidar E, et al. Circulating BMP10 Levels Associate With Late Postoperative Atrial Fibrillation and Left Atrial Endomysial Fibrosis. JACC Clin Electrophysiol. 2024;10(7 Pt 1):1326–40. 38639699 10.1016/j.jacep.2024.03.003

[9] Hennings E, Aeschbacher S, Coslovsky M, Paladini RE, Meyre PB, Voellmin G et al. Association of bone morphogenetic protein 10 and recurrent atrial fibrillation after catheter ablation. Europace. 2023;25(6):euad149. 10.1093/europace/euad149 PMC10265951 37314197

[10] Chua W, Khashaba A, Canagarajah H, Nielsen JC, di Biase L, Haeusler KG et al. Disturbed atrial metabolism, shear stress, and cardiac load contribute to atrial fibrillation after ablation: AXAFA biomolecule study. Europace. 2024;26(2):euae028. 10.1093/europace/euae028 PMC10873713 38266130

[11] Meyre PB, Aeschbacher S, Blum S, Voellmin G, Kastner PM, Hennings E, et al. Biomarkers associated with rhythm status after cardioversion in patients with atrial fibrillation. Sci Rep. 2022;12(1):1680. 35102265 10.1038/s41598-022-05769-9 PMC8803959

[12] Hennings E, Blum S, Aeschbacher S, Coslovsky M, Knecht S, Eken C, et al. Bone Morphogenetic Protein 10-A Novel Biomarker to Predict Adverse Outcomes in Patients With Atrial Fibrillation. J Am Heart Assoc. 2023;12(6):e028255. 36926939 10.1161/JAHA.122.028255 PMC10111531

[13] Gkarmiris KI, Lindback J, Alexander JH, Granger CB, Kastner P, Lopes RD, et al. Repeated Measurement of the Novel Atrial Biomarker BMP10 (Bone Morphogenetic Protein 10) Refines Risk Stratification in Anticoagulated Patients With Atrial Fibrillation: Insights From the ARISTOTLE Trial. J Am Heart Assoc. 2024;13(7):e033720. 38529655 10.1161/JAHA.123.033720 PMC11179770

[14] Hijazi Z, Benz AP, Lindback J, Alexander JH, Connolly SJ, Eikelboom JW, et al. Bone morphogenetic protein 10: a novel risk marker of ischaemic stroke in patients with atrial fibrillation. Eur Heart J. 2023;44(3):208–18. 36380569 10.1093/eurheartj/ehac632 PMC9839419

[15] Van Gelder IC, Rienstra M, Bunting KV, Casado-Arroyo R, Caso V, Crijns H, et al. 2024 ESC Guidelines for the management of atrial fibrillation developed in collaboration with the European Association for Cardio-Thoracic Surgery (EACTS). Eur Heart J. 2024;45(36):3314–414. 39210723 10.1093/eurheartj/ehae176

[16] Suthahar N, Yap SC, de Vries AAF, Damman K, Fabritz L, Bakker SJL, et al. Bone morphogenetic protein 10: clinical correlates and risk of incident atrial fibrillation. Am J Physiol Heart Circ Physiol. 2025;329(5):H1414–21. 41071685 10.1152/ajpheart.00659.2025 PMC7618395

[17] Habibi Z, Verhaert DVM, Betz K, Hermans BJM, Winters J, Philippens SAM, et al. Association of atrial fibrillation burden and clinical profile with blood biomarkers: Results from the ISOLATION Ablation Cohort. Heart Rhythm O2. 2025;6(5):661–70. 40496590 10.1016/j.hroo.2025.02.017 PMC12147586

[18] Chen L, Luo S, Feng M, Chandra A, Baizabal-Carvallo JF, Mofatteh M, et al. Decoding the link between ischemic stroke and myocardial infarction: a pathophysiological and clinical perspective. Eur J Med Res. 2025;30(1):984. 41102842 10.1186/s40001-025-03221-y PMC12533319

[19] Naylor R, Rantner B, Ancetti S, de Borst GJ, De Carlo M, Halliday A, et al. Editor’s Choice - European Society for Vascular Surgery (ESVS) 2023 Clinical Practice Guidelines on the Management of Atherosclerotic Carotid and Vertebral Artery Disease. Eur J Vasc Endovasc Surg. 2023;65(1):7–111. 35598721 10.1016/j.ejvs.2022.04.011

[20] Mazzolai L, Teixido-Tura G, Lanzi S, Boc V, Bossone E, Brodmann M, et al. 2024 ESC Guidelines for the management of peripheral arterial and aortic diseases. Eur Heart J. 2024;45(36):3538–700. 39210722 10.1093/eurheartj/ehae179

[21] Munter Jd. In: Statistics, DoRa, editors. Statistical register’s production and quality National Cause of Death Register. Stockholm: National Board of Social Affairs and Health; 2022.

[22] Bosco E, Hsueh L, McConeghy KW, Gravenstein S, Saade E. Major adverse cardiovascular event definitions used in observational analysis of administrative databases: a systematic review. BMC Med Res Methodol. 2021;21(1):241. 34742250 10.1186/s12874-021-01440-5 PMC8571870

[23] Halvorsen S, Mehilli J, Cassese S, Hall TS, Abdelhamid M, Barbato E, et al. 2022 ESC Guidelines on cardiovascular assessment and management of patients undergoing non-cardiac surgery. Eur Heart J. 2022;43(39):3826–924. 36017553 10.1093/eurheartj/ehac270

[24] Thybring G. National guidelines for pre-analytical handling of liquid-based samples. Stockholm, Sweden; 2020. Report No.: Y2-BIS2020-R.

[25] Bonati LH, Kakkos S, Berkefeld J, de Borst GJ, Bulbulia R, Halliday A, et al. European Stroke Organisation guideline on endarterectomy and stenting for carotid artery stenosis. Eur Stroke J. 2021;6(2):I–XLVII. 34414302 10.1177/23969873211012121 PMC8370069

[26] Aguilar M, Macle L, Ditac G, Benali K, Honfo SH, Khairy P, et al. Atrial fibrillation burden is underestimated by non-invasive monitoring: the CIRCA-DOSE trial. Eur Heart J. 2026;47(2):266–8. 40972515 10.1093/eurheartj/ehaf647

[27] Bjorkenheim A, Fengsrud E, Blomstrom-Lundqvist C. Catheter ablation of symptomatic atrial fibrillation: Sex, ethnicity, and socioeconomic disparities. Heart Rhythm O2. 2022;3(6Part B):766–70. 36588997 10.1016/j.hroo.2022.07.008 PMC9795262

